# Pre-exposure prophylaxis (PrEP) uptake and service delivery adaptations during the first wave of the COVID-19 pandemic in 21 PEPFAR-funded countries

**DOI:** 10.1371/journal.pone.0266280

**Published:** 2022-04-05

**Authors:** Michael Kerzner, Anindya K. De, Randy Yee, Ryan Keating, Gaston Djomand, Sharon Stash, Sangeeta Rana, Allison Kimmel, Robyn Eakle, Sara Klucking, Pragna Patel, on behalf of the PEPFAR HIV Pre-exposure Prophylaxis Collaborators

**Affiliations:** 1 Science Unit, Center for State, Tribal, Local, and Territorial Support, Centers for Disease Control and Prevention, Atlanta, GA, United States of America; 2 Division of Global HIV and TB, Center for Global Health, Centers for Disease Control and Prevention, Atlanta, GA, United States of America; 3 Department of International HIV Prevention and Testing, Walter Reed Army Institute of Research, Silver Spring, MD, United States of America; 4 Bureau for Global Health, Office of HIV/AIDS, United States Agency for International Development, Washington, DC, United States of America; 5 Office of the U.S. Global AIDS Coordinator and Health Diplomacy, U.S. Department of State, Washington, DC, United States of America; Beth Israel Deaconess Medical Center/Harvard Medical School, UNITED STATES

## Abstract

**Background:**

Mitigation measures for the first wave of the COVID-19 pandemic and burden on health systems created challenges for pre-exposure prophylaxis (PrEP) service delivery. We examined PrEP uptake in PEPFAR programs before and after the start of the COVID-19 pandemic.

**Methods:**

We studied two PEPFAR program monitoring indicators, using routine Monitoring, Evaluation, Reporting (MER) indicators capturing uptake of PrEP (PrEP_NEW) and overall use of PrEP (PrEP_CURR). We also analyzed descriptive program narratives to understand successes and challenges field teams encountered after the start of the COVID-19 pandemic. To assess changes in coverage of PrEP across 21 countries, we calculated the “PrEP to need ratio” (PnR) using a published methodology. We defined the pre-COVID time period as April 1, 2019 –March 31, 2020 and the COVID time period as April 1, 2020 –March 31, 2021.

**Findings:**

The total number of persons who initiated PrEP increased by 157% from 233,250 in the pre-COVID-19 period compared with 599,935 in the COVID-19 period. All countries, except five, noted significant increases in PrEP uptake. PrEP uptake among adolescent girls and young women (AGYW) increased by 159% from 80,452 AGYW in the pre-COVID-19 period to 208,607 AGYW in the COVID-19 period. There were 77,430 key populations (KP) initiated on PrEP in the pre-COVID-19 period and 209,114 KP initiated in the COVID-19 period (a 170% increase). The PnR increased 214% in the COVID-19 period across all PEPFAR-supported countries. Adaptations, such as multi-month dispensing (MMD) of PrEP; virtual demand creation activities; decentralized, community-based and virtual service delivery, were implemented to maintain PrEP services.

**Conclusions:**

PEPFAR programs continued to maintain and initiate new clients on PrEP despite the challenges posed by the COVID-19 pandemic. Adaptations such as MMD of PrEP and use of technology were vital in expanding service delivery and increasing PrEP coverage.

**Funding:**

This project has been supported by the U.S. President’s Emergency Plan for AIDS Relief.

## Background

By 2020, 37.6 million persons were living with HIV globally and 1.5 million were newly infected that year [1]. To achieve HIV epidemic control, comprehensive HIV prevention efforts are needed. Pre-exposure prophylaxis (PrEP), an antiretroviral medication used to prevent HIV prior to or for ongoing exposure among at-risk persons, is an effective HIV prevention tool [2]. PrEP programs have been slow to scale-up in some countries due to policy and accessibility barriers. In 2016, only nine countries had initiated approximately 100,000 persons on PrEP; four were in Africa: Ethiopia, Senegal, South Africa, and Zimbabwe [3]. Thus, the total number of people who have been enrolled on PrEP has fallen short of the UNAIDS goal of three million persons on PrEP by 2020 [4]. The U.S. President’s Emergency Plan for AIDS Relief (PEPFAR) started implementing PrEP in 2016 and made PrEP a core requirement for programs in 2020 with a target of reaching one million persons by the end of September 2021 with $98 million of dedicated funding. This and other strides made in HIV epidemic control, such as scale-up of antiretroviral therapy, are now threatened by the novel coronavirus disease-19 (COVID-19) pandemic which is caused by severe acute respiratory syndrome coronavirus 2 (SARS CoV-2) [5].

In response to the COVID-19 pandemic, PEPFAR released COVID-19 guidelines for programming and made PrEP an essential service, prioritizing the maintenance of current clients on PrEP during the COVID-19 pandemic [6]. The COVID-19 adaptation recommendations included multi-month dispensing (MMD) of PrEP; decentralized services from clinics to communities including drug delivery; virtual service delivery; use of technology such as short message service for adherence reminders and appointment reminders, and demand creation using no-contact or contact-limited platforms (e.g., social media such as WhatsApp) with the engagement of peers and community leaders [6–9]. Some of these adaptations such as decentralized community-based service delivery models and use of technology for reminders were used in a few countries [7–9]. Given the heterogeneity across countries of the COVID-19 epidemic as well as implementation of PrEP–many programs were nascent–we were unsure how the majority of PEPFAR-supported PrEP programs were impacted by COVID-19 mitigation strategies. We hypothesize that the countries with large, seasoned programs were best poised to maintain service delivery. To understand PrEP use in the context of COVID-19, we examined available data during the COVID-19 pandemic compared with a similar period of time prior to the COVID-19 pandemic for all PEPFAR-supported countries that are currently implementing PrEP. We also described the COVID-19 related mitigation measures and adaptations to PrEP service delivery, including best practices, to maintain programming in selected countries. This analysis aims to understand the extent to which PEPFAR PrEP programs have continued to implement and maintain continuity of services by adapting services to the COVID-19 context.

## Methods

### PrEP implementation in PEPFAR countries

In 34 PEPFAR-supported countries, PrEP is offered to at-risk clients, as defined by protocols based on the World Health Organization (WHO) and national guidelines which includes HIV risk assessment and adherence counseling [10]. The HIV risk screening occurs in both community and health facility settings and is adapted to country-specific HIV epidemiology. Once initiated, clients are counseled on adherence which assesses their ability to take PrEP as prescribed. PrEP should be initiated as a part of combination prevention strategies, which include testing and treatment for sexually transmitted infections, condoms, family planning counseling, contraception, and mental health counseling, as available. Follow-up, including HIV testing and risk assessment and counseling, occurs every three months; follow-up of some clients, such as adolescents, also occurs after the first month of PrEP initiation in some programs. Some programs elicited input from potential PrEP clients, such as how to reach certain groups, how best to communicate with them about PrEP, to provide access to PrEP, and to design patient-centered PrEP services to ensure successful delivery.

PrEP implementation in PEPFAR -supported countries was facilitated by special PEPFAR initiatives such as the Determined, Resilient, Empowered, AIDS-free, Mentored, and Safe (DREAMS) program for HIV prevention among adolescent girls and young women (AGYW) [11] and the Key Populations Investment Fund (KPIF) [12]. As defined by PEPFAR, AGYW consist of females aged 15–24 years old [11], and key populations (KP) consist of multiple high risk groups including men who have sex with men (MSM), sex workers (SW), people who inject drugs (PWID), people in prisons and other closed settings, and transgender people [12]. DREAMS, which started in 2016, is implemented in 15 countries: Botswana, Côte D’Ivoire, Eswatini, Haiti, Kenya, Lesotho, Malawi, Mozambique, Namibia, Rwanda, South Africa, Tanzania, Uganda, Zambia, and Zimbabwe. KPIF, which started in 2018, is implemented in 19 PEPFAR regions and countries: Asia region, Guatemala, Côte D’Ivoire, Dominican Republic, Eswatini, Haiti, Kenya, Lesotho, Malawi, Mozambique, Namibia, Nigeria, South Africa, Tanzania, Ukraine, Uganda, West Africa region, Zambia, and Zimbabwe. Both initiatives include PrEP implementation and scale-up as a core component of combination prevention.

### Analytic approach

We describe PrEP uptake (new initiations) and continuation in the context of COVID-19 (April 1, 2020-March 31, 2021) compared with a period of time prior to the COVID-19 pandemic (April 1, 2019-March 31, 2020) for all PEPFAR-supported countries implementing PrEP and reporting data during the two time periods, overall and by country [13]. These dates were chosen because the majority of PEPFAR-supported countries experienced their first wave of COVID-19 in March 2020. The analyses were limited to countries that reported at least 25 clients initiated on PrEP in each quarter for the time periods of these analyses. We also describe PrEP use among AGYW and KP as they are priority populations for PrEP implementation. For this analysis, we used two quantitative Monitoring, Evaluation, and Reporting (MER) indicators developed by PEPFAR, PrEP_NEW and PrEP_CURR. PrEP_NEW is the number of individuals who were newly enrolled on oral PrEP. PrEP_CURR is the number of individuals, inclusive of those newly enrolled, that received oral PrEP to prevent HIV during the reporting period. For select countries, we describe the COVID-19 related mitigation measures and adaptations to PrEP service delivery. Country PEPFAR teams were required to submit descriptive narratives that included data and detailed information about aspects of PrEP programming. These narratives primarily informed PrEP service delivery adaptations in the context of COVID-19. These data were submitted quarterly by PEPFAR-supported countries. This activity was reviewed in accordance with CDC human research protection procedures and was determined to be a non-research public health program activity.

### Qualitative analysis

Two main qualitative datasets were accessed and analyzed: (1) the narrative reports for the MER indicators, PrEP_NEW and PrEP_CURR, and (2) the COVID-19 mitigation strategies and policies. A thematic analysis of the MER narratives was conducted using a combination of inductive and deductive coding processes. Deductive codes were developed through team discussion and review, including prior narrative analyses for the indicators of interest. Example deductive and inductive codes include “COVID-19 Challenges” and “Partnerships” respectively. Coding of the MER narratives was conducted by two of the authors of this paper. The steps followed for the thematic analysis were (1) project staff discussed and identified initial deductive codes; (2) each narrative was read and brief memos written, noting emerging themes or issues; (3) coders and project staff discussed themes from the second step, identifying inductive codes to use in analysis; (4) a formal codebook was constructed of both the deductive and inductive codes; (5) all narratives were then coded, with summative memos written for each narrative by one coder; (6) narratives were iteratively recoded upon developing new inductive codes; (7) main themes were developed from the coded segments and summative memos; visual mapping methods were utilized including MAXMaps during this step (VERBI Software, Berlin, Germany). Project staff selected PrEP datasets for the qualitative analysis from three countries–Kenya, South Africa, Uganda–that were early adopters; part of both DREAMS and KPIF; that had large, interagency PrEP programs in 2019 (Kenya, South Africa, and Uganda accounted for >33% of PrEP initiations by September 2019); and that reported on adaptations in their narratives. All narratives from the three countries were coded and analyzed within the period of study (Kenya (n = 324), South Africa (n = 125), Uganda (n = 128)) using MAXQDA Analytics Pro 2020 (VERBI Software, Berlin, Germany).

The mitigation strategy and policy qualitative dataset consisted of data from the International Task Force’s (ITF) COVID-19 Dashboard within the CDC’s COVID-19 pandemic response and the World Health Organization’s Public Health and Social Measures global data [13]. The COVID-19 mitigation policy dataset was reviewed with descriptions of actions taken being placed into overarching categorical values applied consistently across all three countries. Examples include “school closures”, “point of entry,” and “social gathering”. Additionally, each measure or policy action taken was coded as being “implemented,” “strengthened,” or “eased” for the event date. These events could then contextualize the MER narrative data and then be overlaid with PrEP uptake quantitative data to visually depict PrEP uptake in relation to implementation of COVID-19 mitigation measures.

### Quantitative analysis

We examined both **PrEP uptake**, the number of persons initiating PrEP, in the two time periods and the percentage change in uptake from the pre-COVID-19 period to the COVID-19 period as the primary outcomes of the analysis. Given that the PrEP landscape was different in the COVID-19 period with aspirations to reach one million persons, we estimated PrEP_NEW achievement to understand the contextual relevance of PrEP_NEW results. **PrEP_NEW achievement** was estimated using the ratio of PrEP_NEW divided by the corresponding PrEP target (i.e, goal) for that time period. Annual targets were divided evenly across each quarter in the time periods examined. (see Appendix). Of persons who received PrEP as reflected by PrEP_CURR, we examined the disaggregate of the indicator for persons with three-month follow-up HIV testing during the time period examined as a proxy for continuation in the PrEP program, but not adherence. Because targets for PrEP_CURR were not available for FY2019, calculations for achievements were not conducted. Results are reported and calculated at the country-aggregated level and represent data from implementing partners. We conducted z-statistic based statistical tests to determine if the percent changes were statistically significant at a predetermined level. For counts, we used Poisson distribution, and for percentages, we used binomial distribution to formulate the test statistics. Significance of the tests were determined under the assumption of asymptotic normality and the level of significance was set at p-value = 0·05.

We calculated the **PrEP-to-need ratio** (PnR), which is defined as the ratio of the number of new PrEP users to the number of new HIV diagnoses for a given geographic area/or population [14]. PrEP users were defined as persons initiating PrEP during the time period, therefore, we used PrEP_NEW to estimate cumulative PrEP users during the annual time period. As national HIV incidence data were unavailable for these time periods, we used the number of persons with an HIV positive test in the reporting period as a proxy for new HIV diagnoses. This number was calculated using the MER indicator, HTS_TST_POS. We use PnR to assess PrEP coverage in the same geographic region for two different time periods. We used the MER structured dataset from FY21Q2 for all data analyses [15], which were conducted using Excel 2016 (Microsoft Corporation, Seattle, WA). For global COVID-19 data, we used the WHO Coronavirus-19 (COVID-19) Dashboard [16].

## Results

### All populations

**Fig 1:**
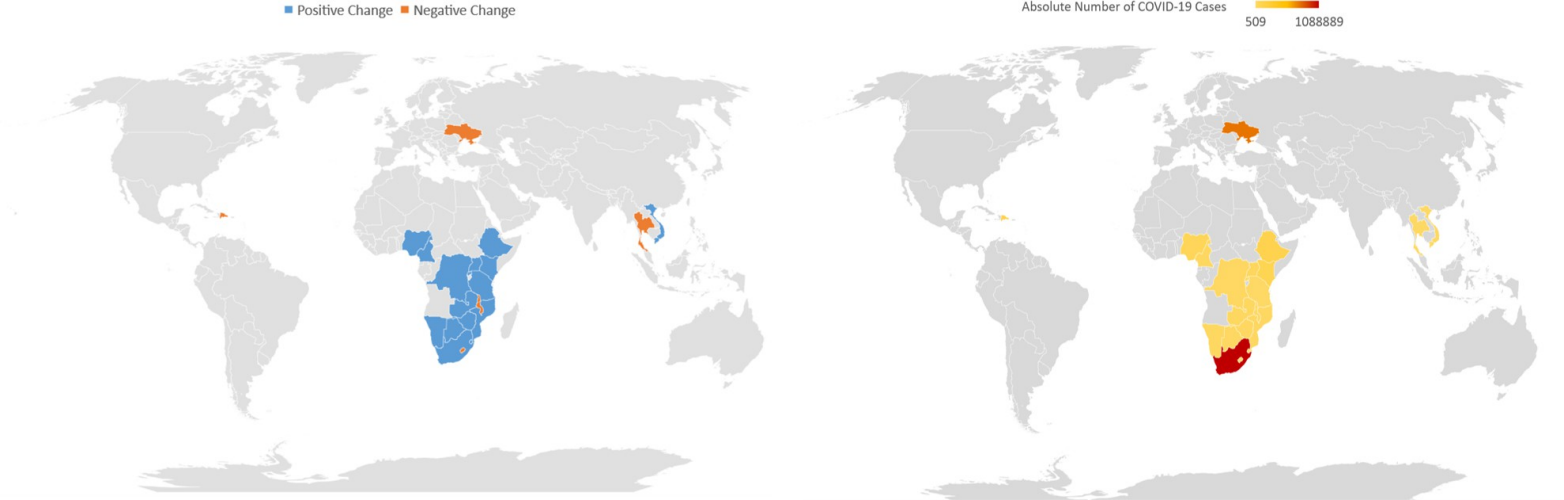
Uptake of pre-exposure prophylaxis in all populations (right) and incidence of COVID-19 (left), by country. a. Percent change in uptake of pre-exposure prophylaxis in all populations from the pre-COVID to COVID periods, by country (right). B. Cumulative COVID-19 cases by country, March 2020 –July 2021 (left). Data source: https://covid19.who.int/ [16]; Maps were created using Microsoft Excel version 2102.

Of the 34 countries examined, 13 countries were excluded from this analysis because they did not meet the designated reporting threshold of 25 clients initiating PrEP each quarter during the time period. In the 21 countries with PEPFAR PrEP programs examined, the total number of persons who initiated PrEP increased from 233,250 in the pre-COVID-19 period to 599,935 in the COVID-19 period, a significant increase of 157% (Table 1). All countries, except five, noted significant increases in PrEP uptake. The number of PrEP clients receiving three-month HIV testing during follow-up increased by 174% in the COVID-19 period compared with the pre-COVID-19 period. Ninety-nine percent of the PrEP_NEW target was achieved in the pre-COVID-19 period compared with 87% in the COVID-19 period; however, the absolute number of PrEP initiators doubled in the COVID-19 period. (Table 1). Most countries noted a positive percent change in PrEP uptake during the COVID-19 period (Fig 1A and 1B).

**Table 1:** New and current preexposure prophylaxis (PrEP) users in PEPFAR-supported countries, by pre-COVID-19 and COVID-19 time periods.

PEPFAR Countries	Time period						Percent change		
	Pre-COVID (April 1, 2019 –March 31, 2020)			COVID-19 (April 1, 2020 –March 31, 2021)					
	PrEP uptake (n)	PrEP uptake achievement (%)	Persons with three-month follow-up	PrEP uptake (n)	PrEP uptake achievement (%)	Persons with three-month follow-up	PrEP uptake^†^	PrEP uptake achievement^§^	Persons with three-month follow-up^§^
All countries	233,250	100	113,129	599,935	90	221,331	157.2	-12.4	95.6
Botswana	1,732	170	1,174	3,829	100	174	121.1	-40.0	-85.2
Cameroon	433	30	260	2,023	50	0*	367.2	80.5	-100.0
Democratic Republic of the Congo	646	70	491	2,217	30	1,017	243.2	-49.3	107.1
Dominican Republic	720	130	993	658	50	725	-8.6	-58.8	-27.0
Eswatini	6,213	160	2,681	11,344	140	982	82.6	-12.9	-63.4
Ethiopia	801	60	704	8,577	100	4,801	970.8	67.3	582.0
Kenya	39,169	110	31,141	59,632	90	54,219	52.2	-23.8	74.1
Lesotho	12,884	80	8,109	12,219	60	13,888	-5.2	-25.6	71.3
Malawi	721	20	284	440	0	180	-39.0	-79.4	-36.6
Mozambique	6,596	90	1,501	21,572	70	2,341	227.0	-19.7	56.0
Namibia	11,144	110	1,496	15,226	80	11,649	36.6	-25.5	678.7
Nigeria	3,435	50	411	84,763	140	29,128	2367.6	210.5	6,987.1
Rwanda	1,621	100	353	7,548	170	8,286	365.6	65.5	2,247.3
South Africa	48,588	70	13,149	146,540	80	16,385	201.6	19.7	24.6
Tanzania	6,045	30	5,460	25,694	20	9,458*	325.0	-27.9	73.2
Thailand	5,754	150	7,294	5,106	90	9,631	-11.3	-42.1	32.0
Uganda	28,532	120	11,208	69,228	110	4,700*	142.6	-9.6	-58.1
Ukraine	1,626	110	439	1,392	70	856	-14.4	-34.0	95.0
Vietnam	7,684	150	4,984	13,276	90	14,595	72.8	-38.5	192.8
Zambia	35,580	250	10,703	86,810	160	20,897	144.0	-33.9	95.2
Zimbabwe	13,326	230	10,294	21,841	140	17,419	63.9	-39.1	69.2

* Indicates possible reporting error.

†All changes are significant at 0.05 level except that of Dominican Republic.

§All changes are significant at 0.05 level.

### Adolescent girls and young women (AGYW)

Overall, 80,452 AGYW initiated PrEP in the pre-COVID-19 period and 208,607 initiated PrEP in the COVID-19 period, reflecting a significant increase of 159%. All countries noted an increase in the number of AGYW initiated on PrEP in the COVID-19 period compared with the pre-COVID period except two (Table 2). Among adolescents aged 15–19 years old, 31,088 initiated PrEP in the pre-COVID-19 period and 76,630 initiated PrEP in the COVID-19 period, a 146% increase. Similarly, among young women aged 20–24 years, 49,364 initiated PrEP in the pre-COVID-19 period and 131,977 initiated PrEP in the COVID-19 period, a 96% increase. Among all AGYW, programs reached 80% of the PrEP_NEW target in the pre-COVID-19 period compared with 70% in the COVID-19 period; however, the absolute number of PrEP initiators more than doubled. Declines in PrEP_NEW target achievement were seen among adolescents aged 15–19 years (-25%) compared with young women aged 20–24 years (-12.3%) (Table 2). Of the 13 countries with DREAMS programs included in this analysis, 11 demonstrated increases in PrEP initiations among AGYW in the COVID-19 period. Six countries had increases in PrEP_NEW achievement in the COVID-19 period compared with the pre-COVID-19 period (Table 2). Cote D’Ivoire and Haiti were the two countries with DREAMS programs excluded from the AGYW-focused analysis because they did not meet the reporting threshold for inclusion.

**Table 2:** New and current preexposure prophylaxis (PrEP) users in PEPFAR-supported countries, by pre-COVID-19 and COVID-19 time periods, among adolescent girls and young women aged 15–24 years.

PEPFAR Countries	Time period				Percent change	
	Pre-COVID (April 1, 2019 –March 31, 2020)		COVID-19 (April 1, 2020 –March 31, 2021)			
	PrEP uptake (n)	PrEP uptake achievement (%)	PrEP uptake (n)	PrEP uptake achievement (%)	PrEP uptake*	PrEP uptake achievement^†^
All countries	80,452	80	208,607	70	159.3	-17.2
Botswana	972	140	1,976	80	103.3	-45.4
Cameroon	46	20	255	50	454.3	116.4
Democratic Republic of the Congo	129	70	337	30	161.2	-52.3
Dominican Republic	88	120	97	70	10.2	-39.4
Eswatini	1,238	160	3,349	100	170.5	-36.2
Ethiopia	423	130	4,367	220	932.4	73.8
Kenya	11,955	120	17,357	80	45.2	-34.3
Lesotho	6,068	190	5,424	80	-10.6	-56.3
Malawi	432	30	168	0	-61.1	-88.7
Mozambique	3,001	150	7,495	60	149.8	-57.8
Namibia	5,169	100	8,198	100	58.6	0.7
Nigeria	298	20	9,877	110	3,214.4	402.0
Rwanda	289	70	4,081	170	1,312.1	134.2
South Africa	28,568	50	83,266	60	191.5	7.0
Tanzania	2,038	30	10,383	20	409.5	-32.1
Uganda	8,519	130	21,199	230	148.8	81.8
Vietnam	203	60	357	40	75.9	-23.7
Zambia	6,381	250	20,538	120	221.9	-53.8
Zimbabwe	4,635	230	9,883	210	113.2	-7.9

*All changes are significant (p<0.05) except that of Dominican Republic.

†All changes are significant except for Namibia.

### Key populations (KP)

Overall, there were 77,430 KP initiated on PrEP in the pre-COVID-19 period and 209,114 initiated on PrEP in the COVID-19 period, reflecting a significant increase of 170%. All countries noted an increase in the number of KP initiated on PrEP in the COVID-19 period compared with the pre-COVID period except three: Dominican Republic (-16%), Thailand (-13%), and Ukraine (-22%). Among female sex workers (FSW), 43,552 initiated PrEP in the pre-COVID-19 period and 125,103 initiated PrEP in the COVID-19 period, a 187% increase. Similarly, among men who have sex with men (MSM), 28,940 initiated PrEP in the pre-COVID-19 period and 63,710 initiated PrEP in the COVID-19 period, a 120% increase. Among all KP, programs reached 100% of PrEP_NEW achievement in the pre-COVID-19 period compared with 120% in the COVID-19 period; similar to AGYW, the absolute number of PrEP initiators more than doubled for KP (Table 3). Increases in PrEP_NEW achievement were largely seen in the African countries, specifically Malawi, South Africa, Zambia, Uganda, Ethiopia, Namibia, Lesotho, and Nigeria. All countries with increases in PrEP_NEW achievement, except Ethiopia, were participating in KPIF activities. However, nine countries (Botswana, Dominican Republic, Kenya, Rwanda, Tanzania, Thailand, Ukraine, Vietnam, Zimbabwe) with KPIF programs had decreases in PrEP_NEW achievement.

**Table 3:** New and current preexposure prophylaxis (PrEP) users in PEPFAR-supported countries, by pre-COVID-19 and COVID-19 time periods, among key populations*.

PEPFAR Countries	Time period				Percent change	
	Pre-COVID (April 1, 2019 –March 31, 2020)		COVID-19 (April 1, 2020 –March 31, 2021)			
	PrEP uptake (n)	PrEP uptake achievement (%)	PrEP uptake (n)	PrEP uptake achievement (%)	PrEP uptake^†^	PrEP uptake achievement^†^
All countries	77,430	100	209,114	120	170.1	28.6
Botswana	901	220	1,765	160	95.9	-27.4
Dominican Republic	718	120	602	90	-16.2	-31.1
Ethiopia	636	60	7,011	160	1,002.4	151.4
Kenya	5,818	100	15,636	80	168.8	-20.7
Lesotho	1,024	40	1,662	150	62.3	262.2
Malawi	366	20	1,199	30	227.6	22.5
Namibia	1,239	50	3,859	160	211.5	186.9
Nigeria	2,237	50	49,358	250	2,106.4	399.3
Rwanda	1,513	330	4,358	170	188.0	-49.3
South Africa	15,310	60	21,618	70	41.2	25.0
Tanzania	4,547	100	16,035	50	252.7	-54.3
Thailand	5,744	150	5,017	90	-12.7	-43.0
Uganda	15,847	110	38,891	270	145.4	138.3
Ukraine	1,108	70	869	60	-21.6	-14.3
Vietnam	6,184	130	11,780	90	90.5	-30.3
Zambia	5,076	130	16,529	220	225.6	71.9
Zimbabwe	9,162	300	12,925	200	41.1	-34.7

* Key populations consist of sex workers, men who have sex with men, people who inject drugs, prisoners, and transgender persons.

†All changes are significant (p<0.05).

### COVID-19 mitigation measures in three select countries

**Fig 2:**
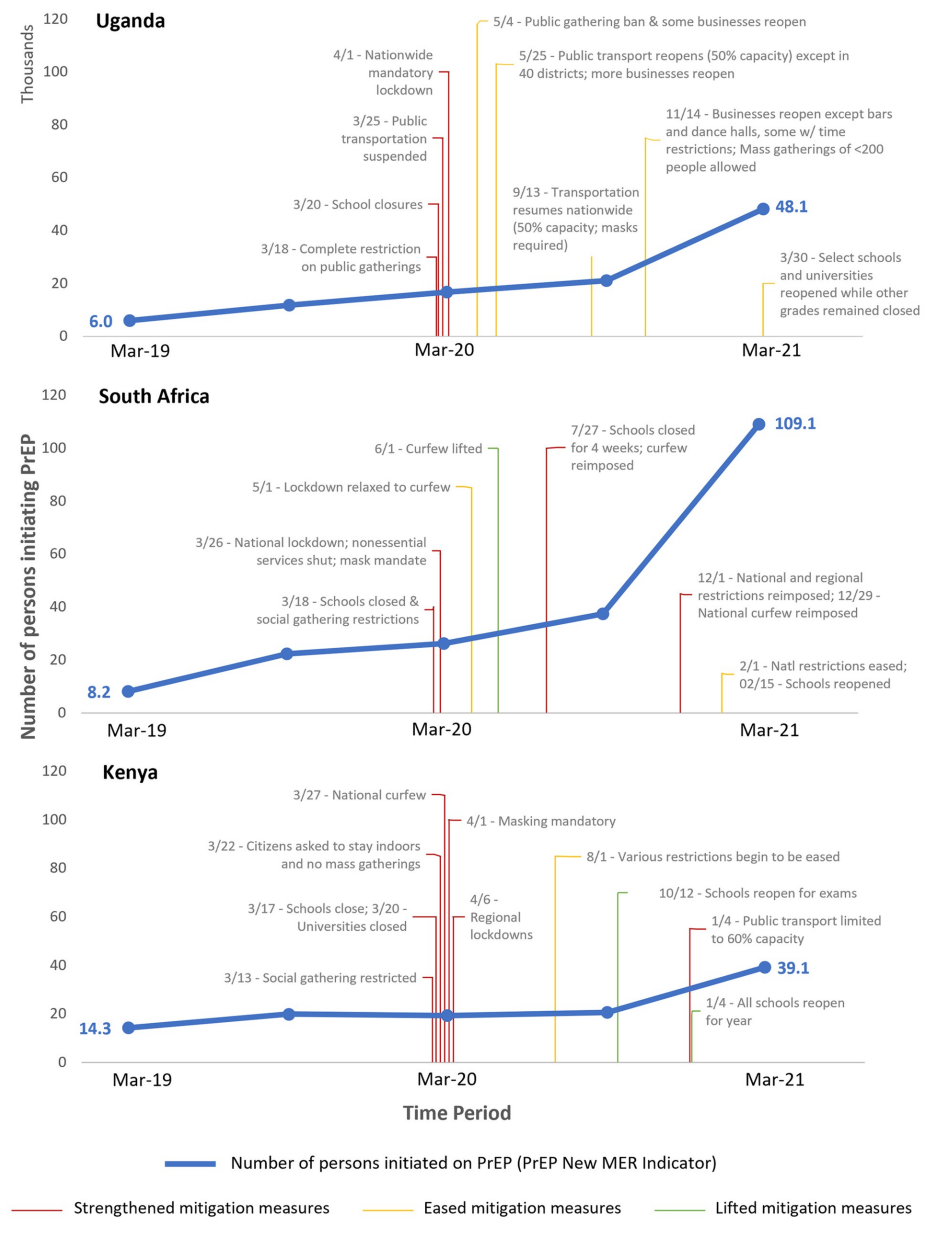
Pre-exposure prophylaxis (PrEP) uptake, by country and by reporting time period, with select COVID-19 mitigation strategies.

An analysis of the COVID-19 mitigation strategy and policy data showed that the first COVID-19 mitigation policies were implemented in late January 2020 and were related to screening of incoming travelers for SARS-CoV-2 infection in Kenya, Uganda, and South Africa. Additional commonly implemented strategies included restrictions on public or social gatherings, closure of schools and universities, lockdowns, curfews, border closures, and travel restrictions; although timing of implementation and duration varied. COVID-19 mitigation efforts varied regionally, or even by city within each country (Fig 2). As such, the implications for programming can vary across programs within a given country.

Several challenges to PrEP programming stemmed directly from COVID-19 mitigation strategies or policies. Some challenges noted in the MER narrative reports, particularly in regions of South Africa, were due to not having current contracts in place for PrEP programming, which prevented specific programs from reserving “essential services” status under regional and national authorities. Many countries faced extended periods of lockdowns and/or curfews, restricting the movements of clients and limiting access to PrEP programming. PrEP health care staff were unavailable due to widespread worker strikes, due to COVID-19 reassignment, or COVID-19 quarantine and isolation protocols, which were reported most widely in Kenya and South Africa. Community delivery channels such as safe spaces and drop-in centers were closed as these were considered non-essential clinical services. School closure limited access to AGYW.

### PrEP program adaptations and best practices

As reported in the MER narratives, many adaptations such as MMD of PrEP, virtual demand creation, and community and/or virtual service delivery were implemented to maintain access to service delivery and are considered best practices (Table 4) [7–9]. MMD reduced the need for frequent in-person appointments with clients and ensured clients had the medication they required. Programs reported different approaches to decentralize services, including the utilization of mobile units such as vans to provide PrEP and other services in the community and using virtual service delivery by holding PrEP initiation appointments and adherence counseling with clients over the phone or sending prescriptions and/or appointment reminders via WhatsApp. Programs from all three countries reported leveraging media for demand creation or community education for PrEP services. They also heavily utilized new media such as social media, video streaming platforms, and other internet-based means to engage their communities. Technology was reported to be key in maintaining all facets of PrEP programming.

**Table 4:** Summary of pre-exposure prophylaxis program adaptations by country and by population.

Country & Program Area	PrEP Program Adaptations	Date of Reported Activity*		
Kenya	Adaptations consistent across two-three countries in bold	FY20 Q2	FY20 Q4	FY21 Q1
*Management and Policy*	**Virtual staff training**			
	Prioritization of cases			
	Public-private sector **partnerships**			
	Rearranged workflow and schedules implemented for COVID-19			
	Strategic information generation/coordination/implementation and standardized reporting			
	**Automation** (records management & tracking; service delivery facilitation including appointment reminders, prescription refills, follow-up/check-in calls)			
*Service Delivery*	**Decentralization of services**			
	**Multi-Month Dispensing (MMD) of PrEP**			
	**Integrate PrEP with other services or delivery points**			
*Communication and Outreach*	**PrEP awareness campaigns**			
	**COVID-19 PrEP information, education, and communication (IEC) materials**			
	**Virtual engagement** (forums, demand creation)			
	Individual education and mobilization adhering to COVID-19 prevention practices			
**South Africa**		**FY20 Q2**	**FY20** **Q4**	**FY21** **Q1**
*Management and Policy*	New sites identified for PrEP			
	Doctor networks identified and contracted for service implementation			
	**Virtual training or upskilling staff**			
	Retrained staff for remote call center			
	Community venues identified as alternative to closed/restricted schools w/COVID-19 measures			
	**Automation of records** and follow up via Emergency Call Center			
	**New partnerships** developed			
*Service Delivery*	Scheduling initial PrEP appointments upon request by potential client once lockdown lifted			
	**Decentralization of service delivery:** Mobile units for treatment, appointments, PrEP maintenance, specimen collection, other services			
	Appointment and treatment **reminders**			
	**Decentralization of service delivery:** Community-based services provided			
	Extended service hours upon lifting of lockdowns			
	**Decentralization of service delivery:** PrEP delivery via registered mail			
	Motivational interviewing & links to services			
	**Multi-Month Dispensing (MMD) of PrEP–dispensing three months at a time**			
	Reduced group size for program sessions adhering to COVID prevention practices			
	**Decentralization of service delivery;** PrEP home deliveries and service provision (appointments)			
	M-groups/Clinicians at M-groups for follow up appointments			
*Communication and Outreach*	**COVID-19 PrEP IEC materials** developed			
	**Virtual engagement:** Social media education, myth busting, outreach, and promotion			
	Extended Call Centre expansion of staff and services			
	Call center for follow up & obtaining PrEP commitments for uptake upon visits being allowed			
	**Awareness and promotional campaigns** upon ease of COVID restrictions			
	**Virtual engagement:** PrEP campaigns including on social media			
	Key Population (KP) targeted video clips and live interviews over social media			
	Radio coverage			
**Uganda**		**FY20 Q2**	**FY20** **Q4**	**FY21** **Q1**
*Management and Policy*	PrEP commodity tracking			
	Continuous quality improvement site activities			
	Data reviews to identify implementation gaps (reasons for declining PrEP or missed appointments)			
	PrEP technical working group worked with Ministry of Health to revise national PrEP guidelines to provide a more favorable policy environment for AGYW and pregnant/breast-feeding women			
*Service Delivery*	Routine HIV tests at one- and -three-months follow-up			
	**Integration with index testing services**			
	Community distribution points & refills at key population (KP)-friendly drop-in centers in KP hotspots			
	**Multi-Month Dispensing (MMD) of PrEP–three months at a time**			
	Peer leaders providing door-to-door refills			
	Continuation of risk screening tools, client enrollment, counseling, follow-ups, and retesting			
	**Decentralization of service delivery:** Virtual options for client initiations, refills, and check-ins			
	Flexible clinic hours for community refills			
	KP Civil Society Organization (CSO) follow-ups for those who missed appointments			
	Peer support meetings			
	KP CSO delivering refills			
*Communication and Outreach*	Demand creation with CSOs such as peer-led dialogues			
	**Virtual engagement:** Phone and SMS reminders for refills			
	Print media used			
	**Awareness and promotional campaigns:** Radio Talk Shows used for demand creation			
	Site outreach and client referrals			
	**Virtual engagement:** Social media use including WhatsApp groups			

**Acronyms** = KP: Key population, FY: Fiscal year, MMD: Multi-Month Dispensing, CSO: Civil-society Organization, AGYW: Adolescent girls and young women

IEC: Information, educational and communication.

**Color coding** = 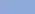: management and policy; 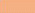: service delivery; 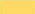: communication and outreach.

*Some reported adaptations as both implemented and planned without further clarity. Adaptations reported in PrEP_CURR & PrEP_NEW narratives are counted twice here.

### PrEP-to-need ratio in the pre-COVID-19 and COVID-19 period

**Fig 3:**
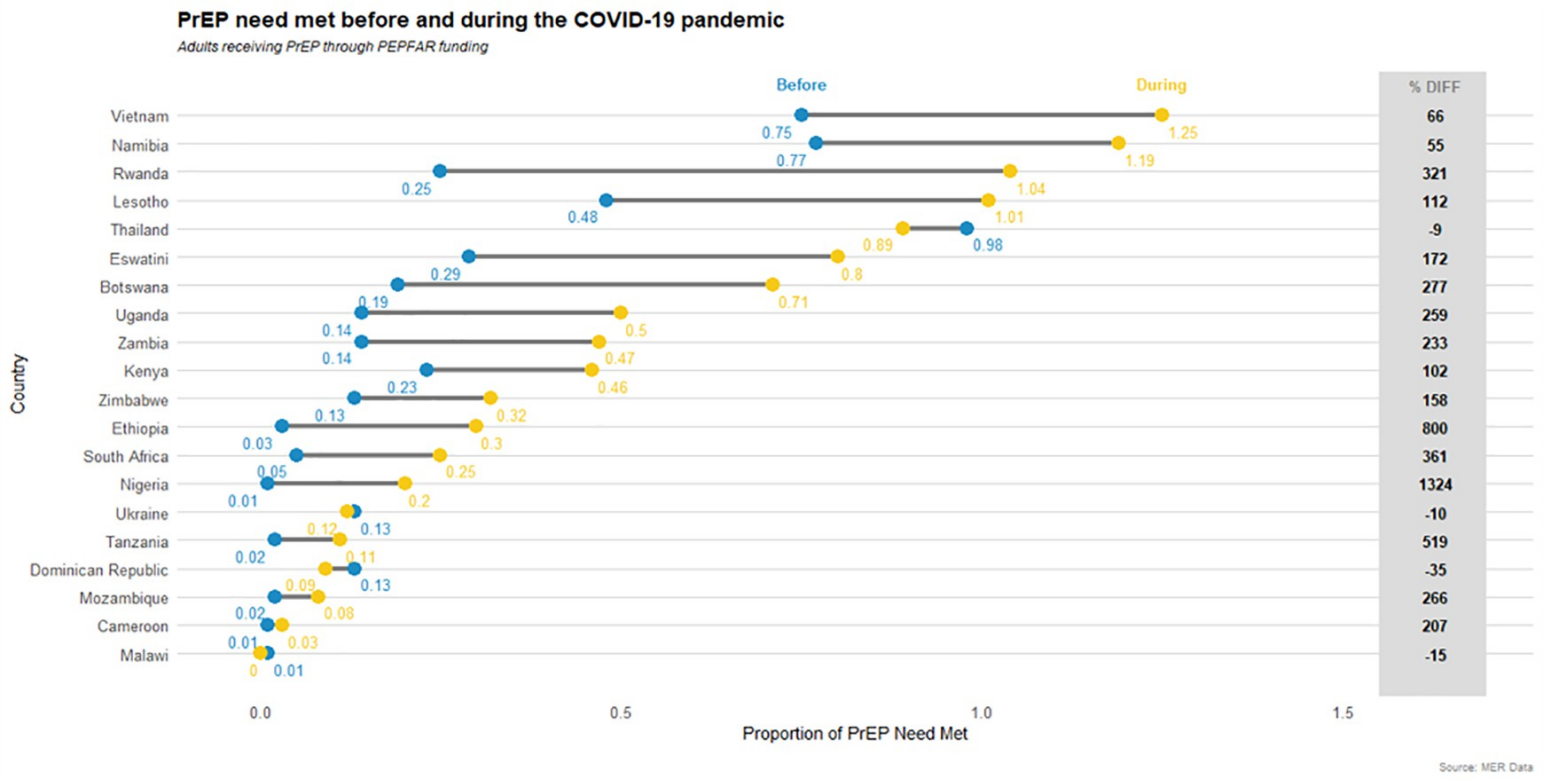
Pre-exposure prophylaxis (PrEP) need met in PEPFAR-supported countries, by pre-COVID-19 and COVID-19 time periods and by country.

The PnR increased 214% in the COVID-19 period across all PEPFAR-supported countries compared with the pre-COVID-19 period. All countries, except the Dominican Republic, Thailand, Malawi, and Ukraine, noted an increased PnR from the pre-COVID-19 time period to the COVID-19 period (Fig 3). In the COVID-19 period, four countries had a PnR≥1.0 (Lesotho (PnR = 1.01), Rwanda (PnR = 1.04), Namibia (PnR = 1.19), and Vietnam (PnR = 1.25). For all other countries, the PrEP-to-need ratio was below 1.0.

## Discussion

Overall, our analyses indicate that several PEPFAR programs were successful in expanding PrEP service delivery despite challenges related to COVID-19 mitigation strategies, particularly lockdown, quarantine, and school closure policies. PEPFAR had ambitious plans to scale up PrEP implementation in 2020 and 2021; thus, countries had plans to reach a substantial number of clients during this time. Recently, there was a significant investment of resources by PEPFAR as well as policy and guideline development by PEPFAR and global partners [17]. This allowed countries to respond to challenges imposed by COVID-19 mitigation strategies and to adapt programs quickly without losing gains in PrEP implementation. As of October 2021, PEPFAR has exceeded achievement of the one million goal set before the pandemic began, initiating 1,593,326 persons on PrEP; PEPFAR PrEP programs support the majority (>80%) of persons who have initiated PrEP worldwide, emphasizing PEPFAR’s major contribution to the global UNAIDS goal [3,4]. Although, declines in achievement were noted initially and expected given COVID-19 mitigation strategies and focusing limited healthcare resources to controlling the COVID-19 pandemic, the absolute number of persons initiating PrEP doubled during the COVID-19 period by adapting programs for virtual and community service delivery. Therefore, best practices from countries with successful adaptations such as decentralized service delivery, virtual approaches to aspects of service delivery including training and demand creation, and multi-month dispensing which have also been proven to be effective in other programs should be disseminated PEPFAR-wide to ensure all countries are able to continue PrEP service delivery as a new standard of care and during future waves of the COVID-19 pandemic [7–9,18–20]. Furthermore, we note that four countries (Lesotho, Rwanda, Namibia, Vietnam) were also able to significantly improve PrEP coverage; these countries may not have been severely impacted by the first wave of the COVID-19 pandemic and some had already made progress towards HIV epidemic control prior to the start of the pandemic [21–23]. Although the 2020 UNAIDS global PrEP target was missed, PEPFAR-supported countries are poised to adapt service delivery and overcome policy barriers for future growth and expansion of PrEP; this will contribute to progress towards the UNAIDS target and ending AIDS by 2030.

Adaptations to PrEP programming, including MMD of PrEP, use of technology, and decentralized, virtual service delivery/engagement, proved to be essential for continued PrEP availability. While some of the adaptations were routinely used in programming prior to the pandemic in a few countries, the COVID-19 pandemic was an opportunity to scale up adaptations for maintenance and expansion of PrEP programs. The use of technology, specifically social media for demand creation and short message service for appointment and medication adherence reminders, were already being incorporated into programs. Community models for PrEP delivery were also being implemented for vulnerable, hard-to-reach populations. This early adoption of adaptations may have contributed to PEPFAR’s ability to ensure continued PrEP delivery during the time of COVID-19. Special initiatives, such as DREAMS and KPIF which included funding for PrEP, may have contributed to innovative approaches to PrEP implementation. In addition, successful countries did not issue policy mandates to halt new PrEP enrollment due to pandemic waves, recognizing access to PrEP for vulnerable populations as an essential service.

PrEP is a core component of DREAMS programming and considerable efforts were focused on PrEP delivery in the pre-COVID period. Because countries were already using technology in their AGYW PrEP programs, increased uptake was noted among AGYW in 11 of 13 countries with DREAMS programs. Given ambitious scale-up plans, the increase in AGYW initiated on PrEP was substantial, yet lower than the aspirational goals, which were three times higher in the COVID-19 period compared with the pre-COVID-19 period. DREAMS is a comprehensive HIV prevention initiative for AGYW that utilizes community-based groups and engagement as well as schools for programming [8]. Therefore, many aspects of DREAMS implementation were halted during the COVID-19 pandemic. In particular, AGYW could not gather in their mentor-led groups, known as safe spaces, which serve as critical access points for interventions. Lack of access to safe spaces and health facilities may explain the declines in uptake in two countries and declines in target achievement; however, the absolute number of AGYW initiating PrEP did increase substantially in 11 countries. As noted in countries with increases in PrEP uptake among AGYW, utilizing virtual platforms and decentralized services might improve PrEP programming during the COVID-19 pandemic such that the ambitious PEPFAR PrEP goals might be achieved in the coming year.

Among KPs, many countries that demonstrated increases in PrEP uptake were participating in KPIF, except Botswana and Ethiopia. KPIF was a central PEPFAR initiative designed to accelerate gains in KP programming in several countries. Its main strategy aimed at strengthening the capacity of KP-led civil society organizations (CSO) to deliver friendly and competent services to KPs. PrEP service delivery underwent pivotal adaptations, as mentioned above and including hot spot drug delivery, during the COVID-19 pandemic, resulting in minimal interruptions of service delivery. Although hot spots were closed in some countries, activities were focused on bringing services closer to KPs and seemed to be more convenient models for people to start and continue PrEP, while adhering to the COVID-19 mitigation strategies. KP-led CSOs were instrumental in the implementation of the PrEP differentiated service delivery models and relentlessly monitored PrEP initiation and continuation among KPs by tracking and maintaining contact with clients. However, many countries participating in KPIF noted decreases in uptake of PrEP among KP and thus continued diligence is warranted to improve scale-up.

Our analysis is not without limitations. We were only able to examine routinely reported MER indicators and PrEP_CURR has not been consistently reported across countries. Although we present data for AGYW and KP separately, there may be overlap that varies across countries. Thirteen countries without PrEP results in FY19 were excluded from our analysis, resulting in an underestimate of uptake in the COVID-19 period. Discrepancies in achievement and absolute number could have arisen due to variation in target setting approaches as well as differing severity of the COVID-19 pandemic across countries. Also, the decline in achievement observed in some countries may be due to other contextual issues, such as hesitancy to roll-out PrEP, that were not necessarily linked to the COVID-19 pandemic, but merely coincidental. The doubling of PrEP initiations suggests that the programs were successful regardless. We are not able to draw associations between countries with the biggest percentage increases in PrEP uptake and which adaptations they implemented due to limited data available in the MER narratives and lack of consistent reporting across all countries. HTS_TST_POS, used in the PnR calculation, reflects the number of individuals that received an HIV positive test in a reporting period, and it may be difficult to de-duplicate the data for repeat testers. Repeat testers likely represent a small proportion of persons who receive a positive test; however, this may vary across countries given different testing strategies and in some, could lead to an overestimation of new HIV diagnoses [24,25]. In addition, many countries observed a decline in HIV testing volume and decline in new HIV positive diagnoses following the first pandemic wave, shrinking the denominator of the PnR [26]. However, declines in testing overall were smaller than increases in PrEP use overall; after July 2020, testing volume and percent of positive HIV tests increased to pre-COVID-19 levels [26]. We did not have access to country-level HIV incidence data from population-based surveys. Furthermore, PnR is an ecological construct; it only represents one moment in time and does not account for changing individual risk.

The PEPFAR PrEP program grew during the period we examined despite the challenges posed by the COVID-19 pandemic, which has inspired innovation and the use of technology for health service delivery [27]. PEPFAR countries implemented pivotal adaptations and differentiated service delivery models as recommended [28]. These adaptations and models proved to be vital for minimal service delivery interruption. To realize the ambitious future PEPFAR PrEP goals, more countries will need to implement these best practices. These approaches will not only sustain vital programming during future waves of the pandemic due to variants [29] but will be essential in reaching all vulnerable populations that may benefit from PrEP.

## Supporting information

S1 Checklist (DOCX)

S1 File (DOCX)

S2 File (XLSX)
