# Supplementary material for: Pre-exposure prophylaxis (PrEP) uptake and service delivery adaptations during the first wave of the COVID-19 pandemic in 21 PEPFAR-funded countries
Source: PLoS One. 2022 Apr 5;17(4):e0266280. doi: 10.1371/journal.pone.0266280 (PMC8982838; doi:10.1371/journal.pone.0266280)
Supplement: S1 File — (DOCX) [file pone.0266280.s002.docx]

**Appendix.** Mathematical formulas

PrEP uptake achievement in pre-COVID-19 period = PrEP_NEW during pre-COVID  divided by PrEP_NEW target for pre-COVID-19 period

PrEP uptake achievement COVID-19 period = PrEP_NEW during COVID-19  divided by PrEP_NEW target for COVID-19 period

(Proportional adjustments were made if the data reported were semi-annual)

Percentage change in PrEP uptake achievement from pre-COVID-19 to COVID-19 period =

100 x (PrEP uptake achievement in COVID period – PrEP uptake achievement in pre-COVID-19 period)

divided by (PrEP uptake achievement in pre-COVID-19 period).

PrEP to Need in pre-COVID-19 period = PrEP_NEW in pre-COVID-19 periods/ HTS_TST_POS in pre-COVID-19 period

PrEP to Need in COVID-19 period = PrEP_NEW in COVID-19 period/ HTS_TST_POS in COVID-19 period

As an illustrative example, a PnR of 2.3 can be interpreted as follows: for each one new HIV diagnosis, there were 2.3 PrEP users. A higher PnR indicates more PrEP users relative to the estimated need, respectively [11].
